# Supplementary material for: The TONSL-MMS22L complex and FANCM form an interdependent complex on chromatin to counter replication stress
Source: bioRxiv. 2025 Mar 10:2025.03.07.642025. Preprint. [Version 1] doi: 10.1101/2025.03.07.642025 (PMC12478395; doi:10.1101/2025.03.07.642025)
Supplement: 1 [file NIHPP2025.03.07.642025v1-supplement-1.pdf]

bioRxiv preprint doi: https://doi.org/10.1101/2025.03.07.642025; this version posted March 11, 2025. The copyright holder for this preprint (which was not certified by peer review) is the author/funder, who has granted bioRxiv a license to display the preprint in perpetuity. It is made available under aCC-BY 4.0 International license.

Supplemental Information

The TONSL-MMS22L complex and FANCM form an interdependent complex on chromatin to counter replication stress

Haixia Zhou, Jiaoyan Yan, Xinlei Cao, Jingfei Zhan, Chen Ling, Yunhui Luo, Zhuang Ma, Yinyying Sun, Peining Song, Weiwei Liu, Lizhu Wang, Jiahuan Li, Althaf Shaik, Marina Bellani, Linqian Wang, Yun Xie, Jing Zhang, Xiangyang Xue, Xian Shen, Michael M. Seidman, Weidong Wang, Zhijiang Yan

bioRxiv preprint doi: https://doi.org/10.1101/2025.03.07.642025; this version posted March 11, 2025. The copyright holder for this preprint (which was not certified by peer review) is the author/funder, who has granted bioRxiv a license to display the preprint in perpetuity. It is made available under aCC-BY 4.0 International license.

Figure S1

A

Flag-FANCM IP-MS

Protein	Unique peptide #
FANCM	72
BLM	28
FANCA	20
FANCE	18
FANCB	15
FANCC	14
MMS22L	14
FANCG	13
TOP3α	13
MCM7	13
FANCF	12
RMI1	11
FAAP100	10
MHF1	10
MHF2	9
TONSL	9
MCM6	9
MCM2	6
MCM4	6
FAAP24	5
FANCL	5
MCM3	5
RMI2	4
PCNA	2
FAAP20	2

B

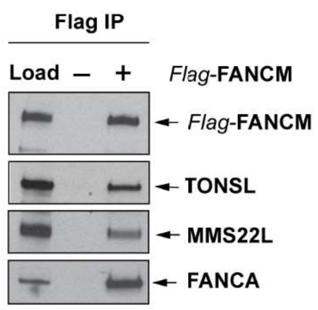

C

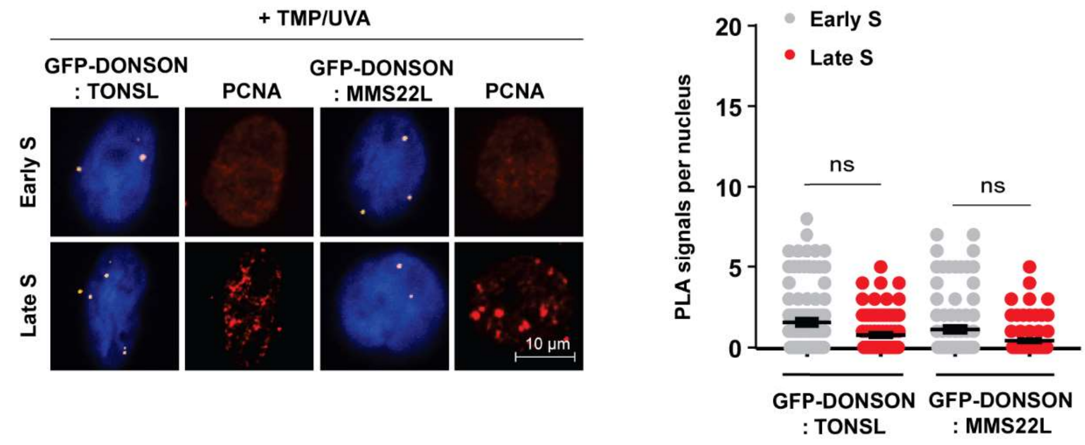

Figure S1 (Related to Figure 1). Replication stress stimulates the FANCM-TONSL-MMS22L complex on chromatin.

bioRxiv preprint doi: https://doi.org/10.1101/2025.03.07.642025; this version posted March 11, 2025. The copyright holder for this preprint (which was not certified by peer review) is the author/funder, who has granted bioRxiv a license to display the preprint in perpetuity. It is made available under aCC-BY 4.0 International license.

(A) We extracted soluble chromatin-bound proteins from native chromatin of HEK293 cells stably expressing Flag-tagged FANCM by treatment with benzonase, a pan nuclease degrading all types of DNA and RNA, and then purified the FANCM complex with a Flag antibody. Mass spectrometry reveals the proteins identified in the FANCM precipitate and the number of unique peptides derived from the corresponding proteins. **(B)** Immunoblotting shows the presences of FANCM, TONSL, MMS22L and FANCA in the precipitate described in (A). HEK293 cells without expression of Flag-FANCM were used as a control. **(C)** *Left panel*: Representative images of the PLA between GFP-DOSON and TONSL or MMS22L in early and late phase cells treated with TMP/UVA. *Right panel*: A graph showing the frequencies of PLA signals described in (C, *Left panel*). Data are mean ± SEM from three biological replicates. Number of nuclei: PLA between GFP-DONSON and TONSL, early S phase cells = 92, late S phase cells = 84; PLA between GFP-DONSON and MMS22L, early S = 86, late S = 87.

bioRxiv preprint doi: https://doi.org/10.1101/2025.03.07.642025; this version posted March 11, 2025. The copyright holder for this preprint (which was not certified by peer review) is the author/funder, who has granted bioRxiv a license to display the preprint in perpetuity. It is made available under aCC-BY 4.0 International license.

Figure S2

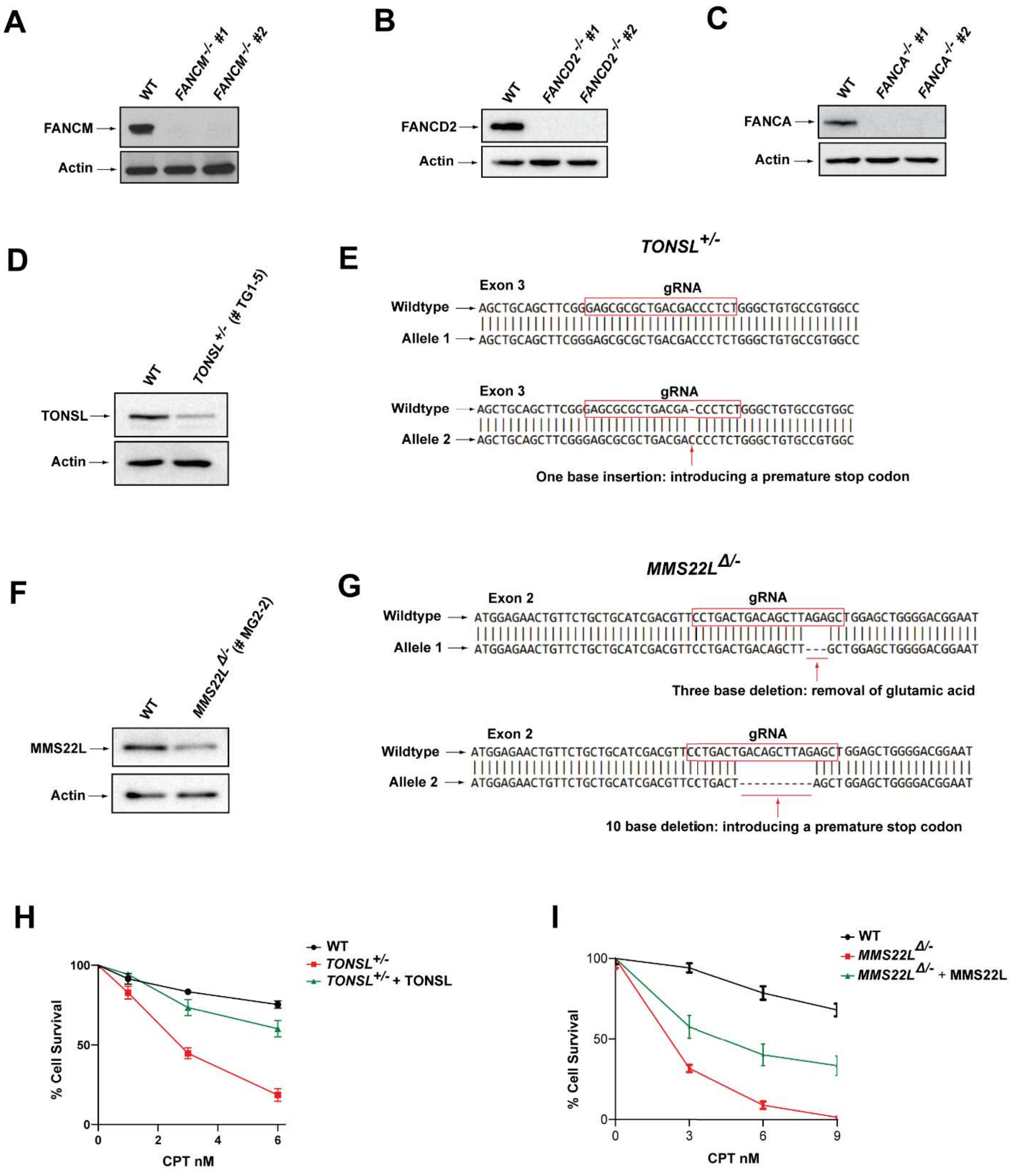

bioRxiv preprint doi: https://doi.org/10.1101/2025.03.07.642025; this version posted March 11, 2025. The copyright holder for this preprint (which was not certified by peer review) is the author/funder, who has granted bioRxiv a license to display the preprint in perpetuity. It is made available under aCC-BY 4.0 International license.

Figure S2 (Related to Figure 2). Both TONSL and MMS22L heterozygotes are hypersensitive to replication stress.

(A) Immunoblotting shows the level of FANCM protein in HeLa wildtype (WT) cells and HeLa cells-derived clones by CRISPR/Cas 9 genome editing using two different gRNA oligos to FANCM locus. Actin was used as a loading control. **(B and C)** As described in (A), except FANCD2 (B) and FANCA (C). **(D and F)** As described in (A), except one gRNA oligo to TONSL locus (D) and one gRNA to MMS22L locus (F). **(E)** Sanger sequencing of the clone TG1-5 showing that one allele is the same as wild type, while the other carries a deletion of one nucleotide within the gRNA-binding sequence that introduces a premature stop codon. These results establish that TG1-5 is a heterozygous knockout clone ($TONSL^{+/-}$). **(G)** As described in (E), except that one allele in the clone MG2-2 carries a deletion of three nucleotides within gRNA-binding sequence, resulting in removal of a glutamic acid; while the other carries a deletion of 10 nucleotides that introduces a premature stop codon. Therefore, MG2-2 is a compound heterozygous knockout clone ($MMS22L^{\Delta/-}$). **(H)** Clonogenic survival assays of HeLa WT cells, $TONSL^{+/-}$ cells and $TONSL^{+/-}$ cells complemented with wildtype TONSL following CPT treatment at the indicated concentrations. Data are mean ± SEM from three independent experiments. Each experiment has triplicate cultures. **(I)** As described in (H), except MMS22L as indicated.

bioRxiv preprint doi: https://doi.org/10.1101/2025.03.07.642025; this version posted March 11, 2025. The copyright holder for this preprint (which was not certified by peer review) is the author/funder, who has granted bioRxiv a license to display the preprint in perpetuity. It is made available under aCC-BY 4.0 International license.

Figure S3

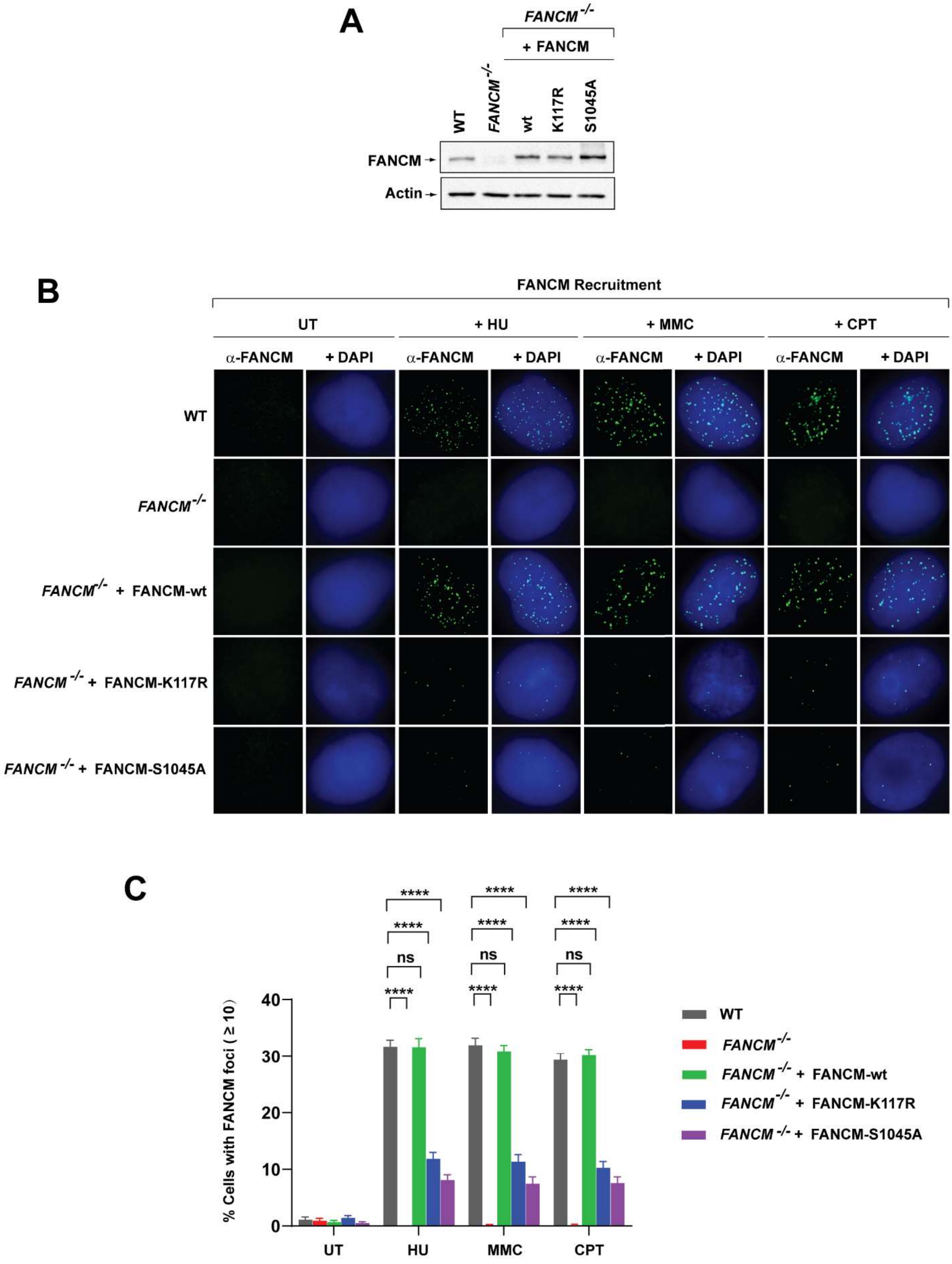

bioRxiv preprint doi: https://doi.org/10.1101/2025.03.07.642025; this version posted March 11, 2025. The copyright holder for this preprint (which was not certified by peer review) is the author/funder, who has granted bioRxiv a license to display the preprint in perpetuity. It is made available under aCC-BY 4.0 International license.

Figure S3 (Related to Figure 2). Both DNA translocase activity and phosphorylation of FANCM are required for its efficient recruitment to stalled and collapsed forks.

(A) Immunoblotting shows the level of FANCM protein in HeLa wildtype (WT) cells, *FANCM*$^{-/-}$ cells and complemented versions with FANCM wildtype (wt), DNA translocase-inactivated mutant FANCM-K117R or phosphorylation-abolished mutant FANCM-S1045A. Actin is an internal control. **(B)** Representative immunofluorescence images showing FANCM nuclear foci in various cell types as described in (A). Cells were treated with HU (2 mM) for 24 h, MMC (60 ng/ml) for 18 h, and CPT (1.5 µM) for 24 h. **(C)** A statistical graph showing the mean values of the percentage of FANCM-foci-positive cells in untreated (UT) and drug-treated cells with standard errors of the mean (SEM) from three independent experiments. A cell containing more than ten foci was considered as foci-positive. At least 200 nuclei were counted for each cell line. Approximate 30% of WT cells revealed more than 10 of large and discrete nuclear foci containing FANCM after exposure to HU, MMC or CPT. In contrast, little few FANCM foci were detected in control cells without drug treatment or in HeLa-derived FANCM null (*FANCM*$^{-/-}$) cells treated with the same drugs. The absence of FANCM foci in *FANCM*$^{-/-}$ cells were all complemented by reintroduction of wildtype FANCM (WT) to the levels equivalent to those in WT cells. Of note, re-expression of FANCM-K117R or FANCM-S1045A restored only ~ 11% and 7.5 % of *FANCM* $^{-/-}$ cells with more than 10 of FANCM foci, respectively, indicating that both DNA translocase activity and phosphorylation of FANCM are required for to its efficient recruitment to stalled and broken forks in human cells.

bioRxiv preprint doi: https://doi.org/10.1101/2025.03.07.642025; this version posted March 11, 2025. The copyright holder for this preprint (which was not certified by peer review) is the author/funder, who has granted bioRxiv a license to display the preprint in perpetuity. It is made available under aCC-BY 4.0 International license.

Figure S4

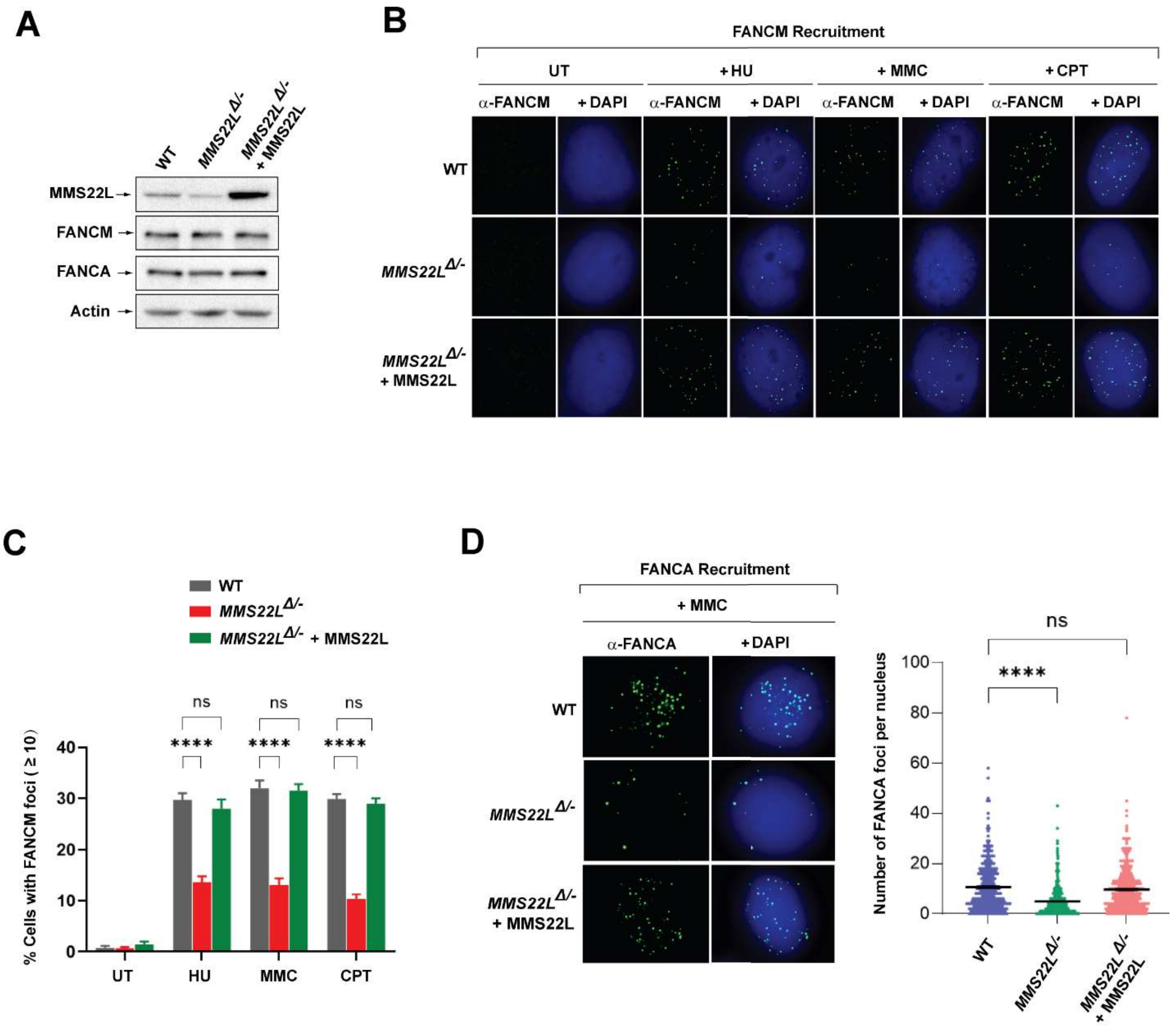

Figure S4 (Related to Figure 2). MMS22L promotes recruitment of FANCM and the FA core complex to stalled and collapsed forks.

(A) Immunoblotting shows the levels of MMS22L, FANCM, FANCA in whole cell lysate from wild-type (WT) HeLa cells, *MMS22L*$^{\Delta/-}$, and *MMS22L*$^{\Delta/-}$ cells complemented with MMS22L. Actin was included as a loading control. **(B)** Immunofluorescence images showing FANCM nuclear foci in various cells described in (A) after treatment with HU (2 mM) for 24 h, MMC (60

bioRxiv preprint doi: https://doi.org/10.1101/2025.03.07.642025; this version posted March 11, 2025. The copyright holder for this preprint (which was not certified by peer review) is the author/funder, who has granted bioRxiv a license to display the preprint in perpetuity. It is made available under aCC-BY 4.0 International license.

ng/ml) for 18 h or CPT (1.5 μM) for 24 hr. **(C)** A statistical graph shows the mean values of the percentage of FANCM-foci-positive cells in untreated (UT) and drug-treated cells from three independent experiments with standard errors of the mean (SEM). A cell containing more than ten foci was considered as foci-positive. At least 200 nuclei were counted for each cell line. The error bars are standard error of the mean (SEM) from three independent experiments. **** represents $P < 0.0001$. "ns" represents non-significant difference. **(D)** As described in (B and C), except a FANCA antibody and MMC were used.

bioRxiv preprint doi: https://doi.org/10.1101/2025.03.07.642025; this version posted March 11, 2025. The copyright holder for this preprint (which was not certified by peer review) is the author/funder, who has granted bioRxiv a license to display the preprint in perpetuity. It is made available under aCC-BY 4.0 International license.

Figure S5

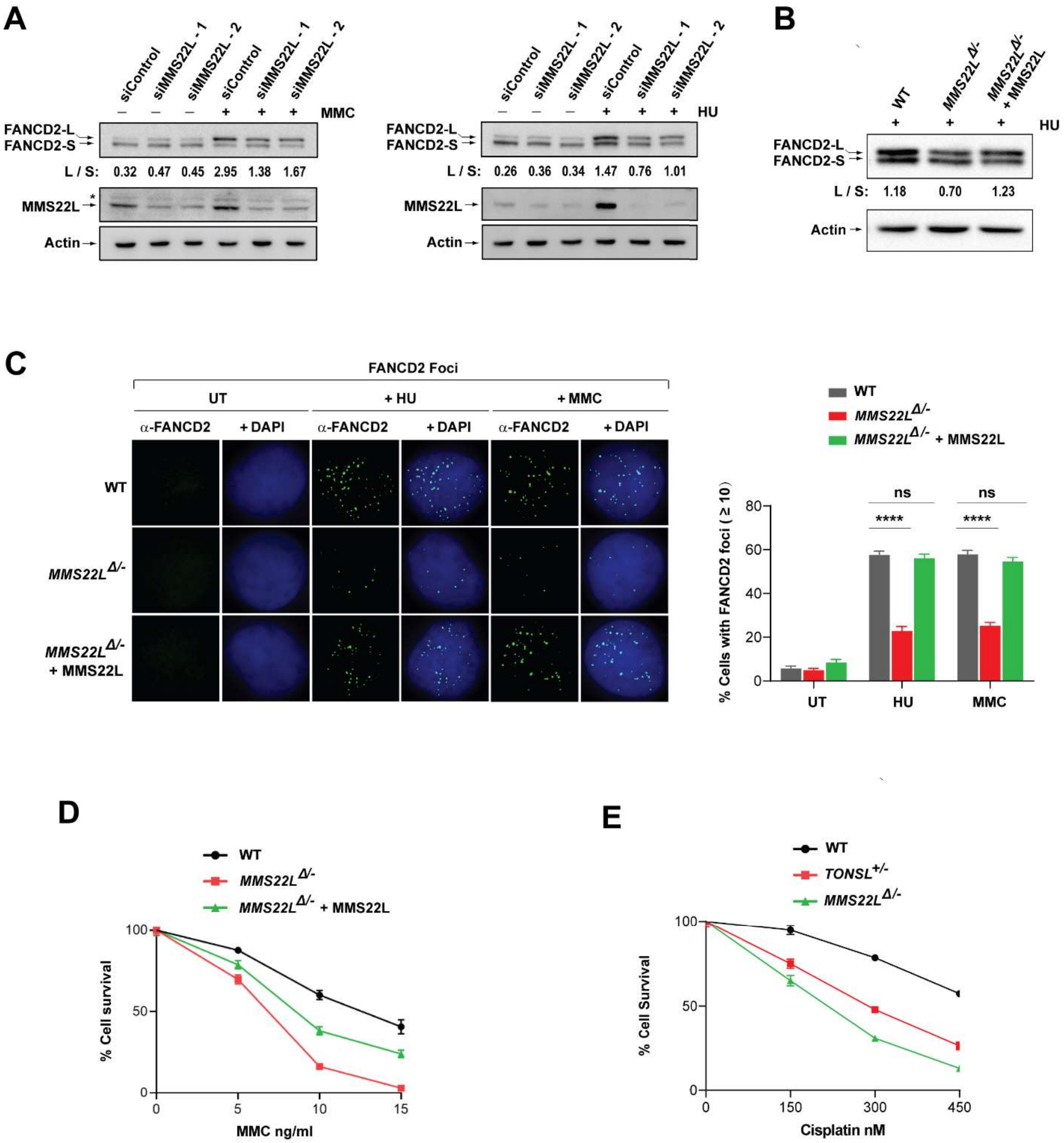

bioRxiv preprint doi: https://doi.org/10.1101/2025.03.07.642025; this version posted March 11, 2025. The copyright holder for this preprint (which was not certified by peer review) is the author/funder, who has granted bioRxiv a license to display the preprint in perpetuity. It is made available under aCC-BY 4.0 International license.

Figure S5 - *continued*

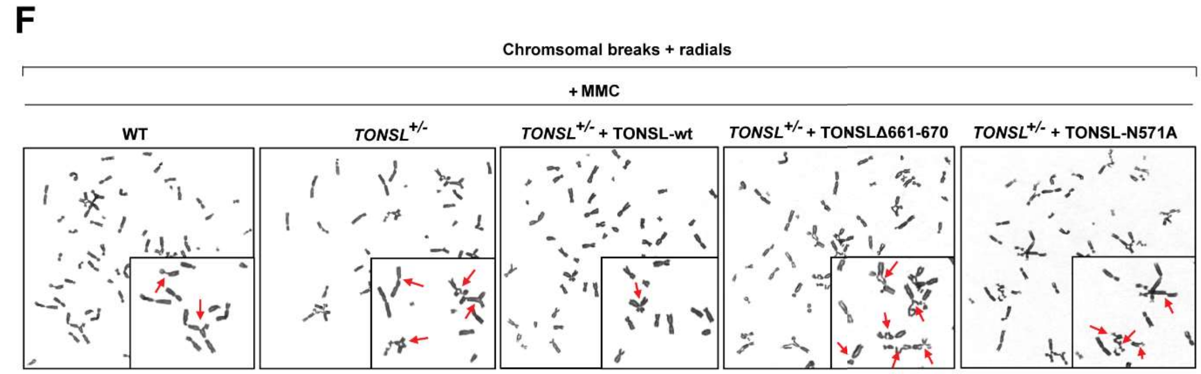

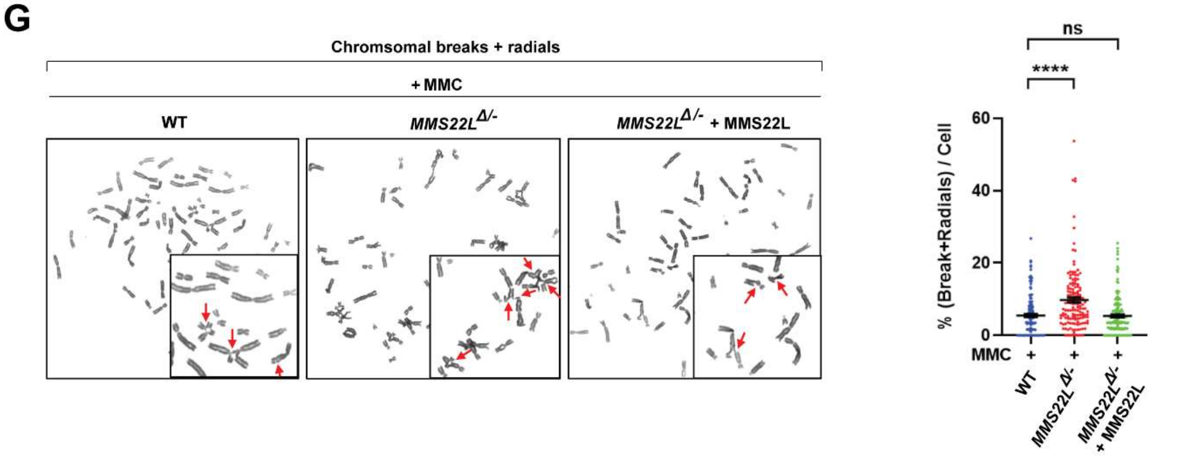

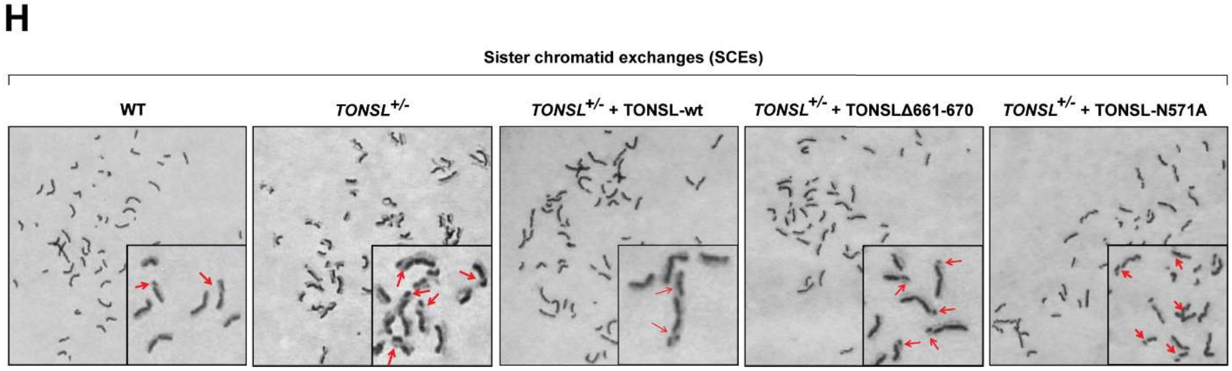

bioRxiv preprint doi: https://doi.org/10.1101/2025.03.07.642025; this version posted March 11, 2025. The copyright holder for this preprint (which was not certified by peer review) is the author/funder, who has granted bioRxiv a license to display the preprint in perpetuity. It is made available under aCC-BY 4.0 International license.

Figure S5 - *continued*

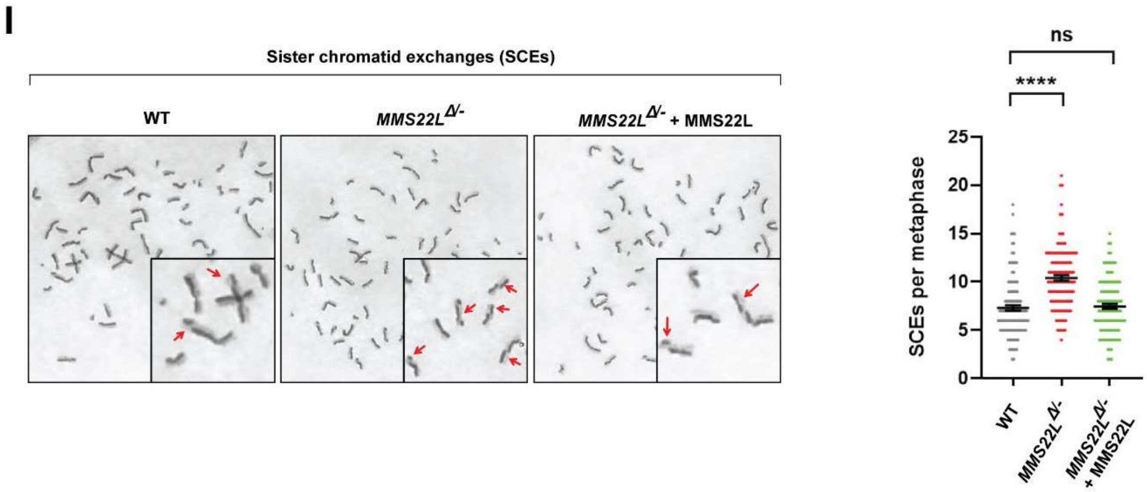

Figure S5 (Related to Figure 3). TONSL-MMS22L complex promotes activation of the FA pathway, ICL repair and SCE suppression.

(A) Immunoblotting shows that HeLa cells depleted of MMS22L have a reduced level of monoubiquitinated FANCD2 in the presence of MMC (60 ng/ml) for 16 h (*Left panel*) or HU (2 mM) for 16 h (*Right panel*). "L" (long) and "S" (short) represent ubiquitinated and non-ubiquitinated forms, respectively. The ratio between long and short forms was obtained using Image J Software and shown below the blots. Actin was used as a loading control. **(B)** As described in (A), except HeLa wildtype cells, *MMS22L*$^{\Delta/-}$ cells and *MMS22L*$^{\Delta/-}$cells complemented with wildtype MMS22L. **(C)** Immunofluorescence images (*Left panel*) and a quantification graph (*Right panel*) showing FANCD2 nuclear foci in various cells described in (B) after treatment with HU (2 mM) for 16 h or MMC (60 ng/ml) for 16 h. Data are mean ± SEM from three independent experiments. **** represents $P < 0.0001$. "ns" represents nonsignificant difference. **(D** and **E)** Clonogenic survival assays of indicated cells following treatment with MMC or cisplatin at the indicated concentrations. **(F)** Representative images showing chromosomal breaks and radial chromosomes in MMC treated various cells as indicated. **(G)** *Left panels:* As described in (F*). Right panels:* A graph showing the number of MMC-induced chromosomal breaks and radial chromosomes in each metaphase cell as indicated. Data are mean ± SEM from three independent experiments. About 50 metaphase cells were counted per each

bioRxiv preprint doi: https://doi.org/10.1101/2025.03.07.642025; this version posted March 11, 2025. The copyright holder for this preprint (which was not certified by peer review) is the author/funder, who has granted bioRxiv a license to display the preprint in perpetuity. It is made available under aCC-BY 4.0 International license.

sample in each experiment. **** represents $P < 0.0001$. "ns" represents nonsignificant difference. **(H)** Representative images showing the number of sister chromatid exchanges in each metaphase cells as indicated. **(I)** *Left panels:* As described in (H). *Right panels:* A graph showing the levels of SCEs in various cells as indicated. Data are mean ± SEM from three independent experiments. About 50 metaphase cells were counted per each sample in each experiment. **** represents $P < 0.0001$. "ns" represents nonsignificant difference.

bioRxiv preprint doi: https://doi.org/10.1101/2025.03.07.642025; this version posted March 11, 2025. The copyright holder for this preprint (which was not certified by peer review) is the author/funder, who has granted bioRxiv a license to display the preprint in perpetuity. It is made available under aCC-BY 4.0 International license.

Figure S6

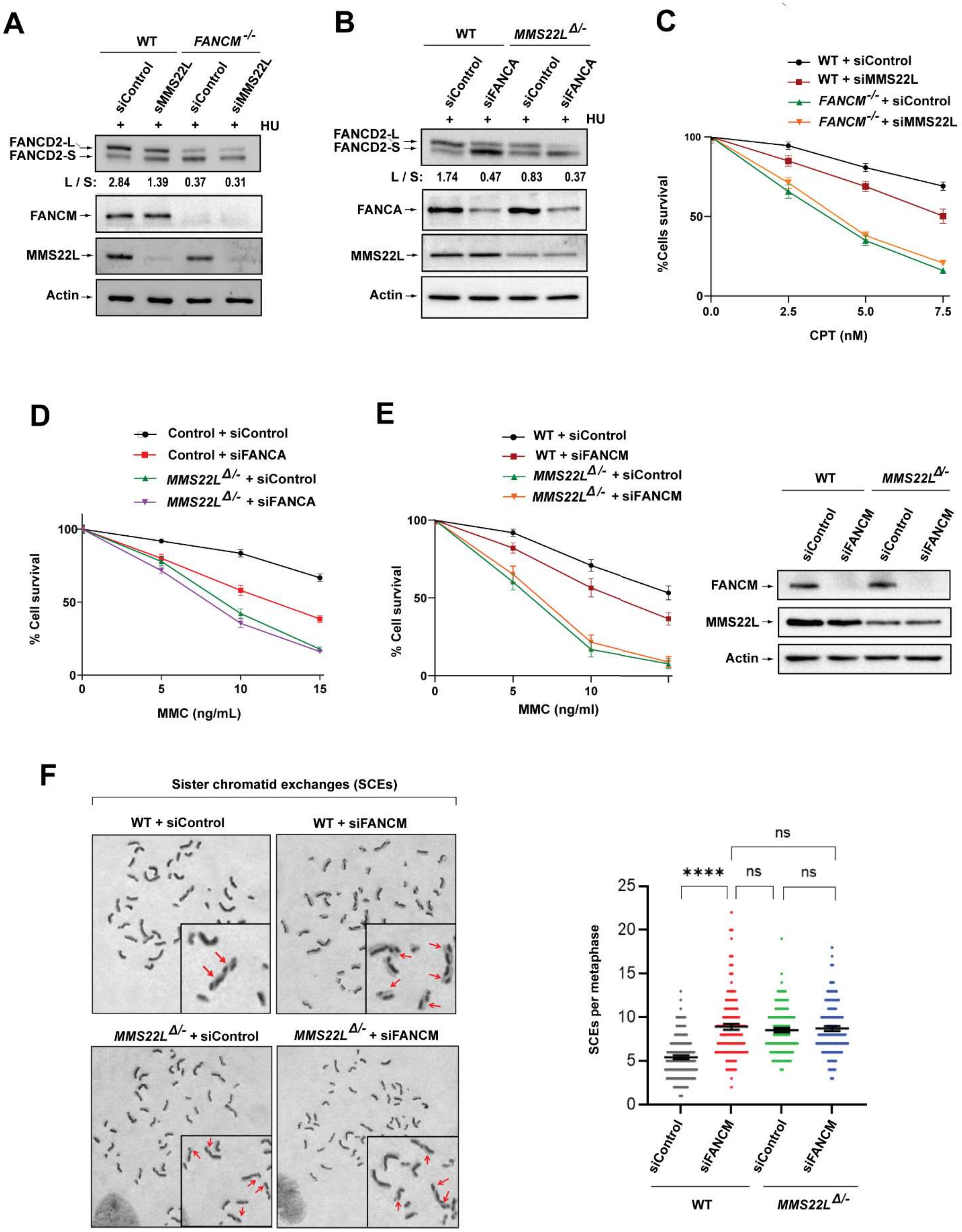

bioRxiv preprint doi: https://doi.org/10.1101/2025.03.07.642025; this version posted March 11, 2025. The copyright holder for this preprint (which was not certified by peer review) is the author/funder, who has granted bioRxiv a license to display the preprint in perpetuity. It is made available under aCC-BY 4.0 International license.

Figure S6 (Related to Figure 4). TONSL-MMS22L works together with FANCM and the FA core complex for FANCD2 monoubiquitination, cellular resistance to replication stress and SCE suppression.

(A) Immunoblotting shows levels of monoubiquitinated and unubiquitinated FANCD2, FANCM, MMS22L in whole cell lysates from various cells as indicated on the top. Cells were treated with HU (2 mM) for 18 h. Actin was included as a loading control. **(B)** As described in (A), excepted FANCA. **(C)** Clonogenic survival assays of various cells as indicated following CPT treatment at the indicated concentrations. Data are mean ± SEM from three independent experiments. Each experiment has triplicate cultures. **(D)** As described in (C), except that MMC was used. **(E)** *Left panels:* As described in (C), except MMC was used. *Right panels:* Immunoblotting shows the levels of FANCM and MMS22L in whole cell lysates from various cells as indicated. Actin was used as a loading control. **(F)** *Left panel:* Representative images showing SCEs (red arrows) in various cells as indicated. *Right panel:* A graph showing the spontaneous SCE levels of various cells as indicated. Data are mean ± SEM from three independent experiments. About 50 metaphase cells were counted per each sample in each experiment. **** represents $P < 0.0001$. “ns” represents nonsignificant difference.

bioRxiv preprint doi: https://doi.org/10.1101/2025.03.07.642025; this version posted March 11, 2025. The copyright holder for this preprint (which was not certified by peer review) is the author/funder, who has granted bioRxiv a license to display the preprint in perpetuity. It is made available under aCC-BY 4.0 International license.

Figure S7

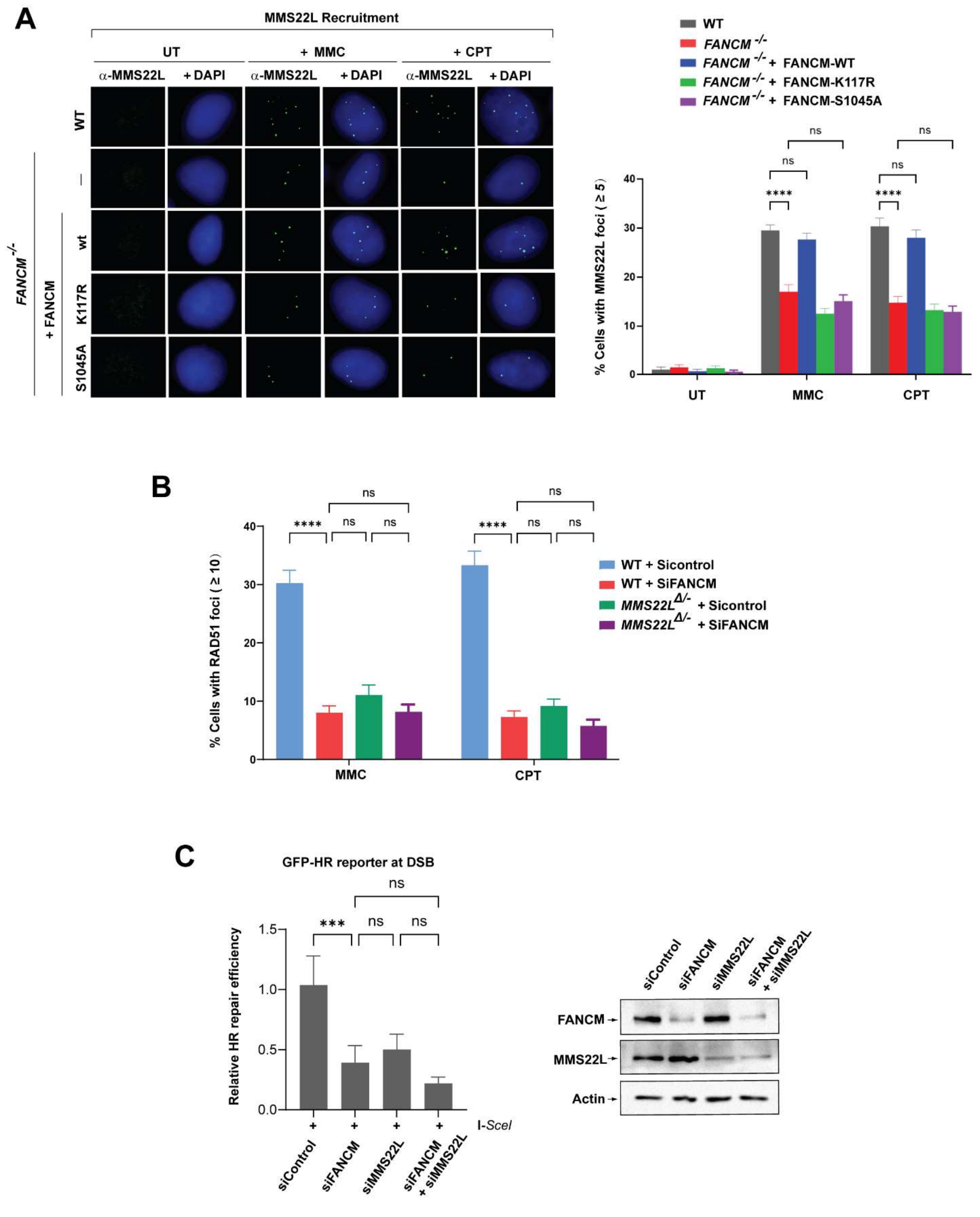

bioRxiv preprint doi: https://doi.org/10.1101/2025.03.07.642025; this version posted March 11, 2025. The copyright holder for this preprint (which was not certified by peer review) is the author/funder, who has granted bioRxiv a license to display the preprint in perpetuity. It is made available under aCC-BY 4.0 International license.

Figure S7 (Related to Figure 6). FANCM and TONSL-MMS22L function together to promote homologous recombination (HR) repair.

(A) Representative images showing MMS22L nuclear foci Immunofluorescence images (*Left panels*) and a quantification graph (*Right panels*) showing MMS22L nuclear foci in various cells after treatment with MMC (60 ng/ml) for 18 h or CPT (1.5 μM) for 24 h. Data are means ± SEM from three independent experiments. **** represents $P < 0.0001$. “ns” represents nonsignificant difference. **(B)** As described in (A), except RAD51 nuclear foci. **(C)** *Left panels:* A quantitation graph showing relative repair efficiency derived from the positive GFP cells in U2OS-DR-GFP-reporter cells transfected with various siRNA oligos as indicated. Data are the mean with SEM from three independent experiments. *** represents $P < 0.001$. “ns” represents nonsignificant difference. *Right panels:* Immunoblotting shows the level of FANCM and MMS22L in whole cell lysates of various cells as indicated. Actin was included as a loading control.
